# Supplementary material for: Emphysema Quantification Using Ultra-Low-Dose Chest CT: Efficacy of Deep Learning-Based Image Reconstruction
Source: Medicina (Kaunas). 2022 Jul 15;58(7):939. doi: 10.3390/medicina58070939 (PMC9317892; doi:10.3390/medicina58070939)
Supplement: Supplementary file 1 [file medicina-58-00939-s001.zip › medicina-1781919-supplementary.pdf]

Supplementary Table S1. Modified medical research council dyspnea scale .

Grade	Degree of breathless related to activities
0	I only get breathless with strenuous exercise
1	I get short of breath when hurrying on the level or walking up a slight hill
2	I walk slower than people of the same age on the level because of breathlessness, or I have to stop for breath when walking on my own pace on the level
3	I stop for breath after walking about 100 m or after a few minutes on the level
4	I am too breathless to leave the house, or I am breathless when dressing or undressing

Supplementary Table S2. Chronic obstructive pulmonary disease assessment test questionnaire.

Questionnaire			Score
I never cough	1 2 3 4 5	I cough all the time	
I have no phlegm in my chest at all	1 2 3 4 5	My chest is completely full of phlegm	
My chest does not feel tight at all	1 2 3 4 5	My chest feels very tight	
When I walk up a hill or one flight of stairs I am not breathless	1 2 3 4 5	When I walk up a hill or one flight of stairs I am very breathless	
I am not limited doing any activities at home	1 2 3 4 5	I am very limited doing activities at home	
I am confident leaving my home despite my lung condition	1 2 3 4 5	I am not at all confident leaving my home because of my lung condition	
I sleep soundly	1 2 3 4 5	I don't sleep soundly because of my lung condition	
I have lots of energy	1 2 3 4 5	I have no energy at all	
Total score			
